# Microbial bioenergetics of coral-algal interactions

**DOI:** 10.7717/peerj.3423

**Published:** 2017-06-21

**Authors:** Ty N.F. Roach, Maria L. Abieri, Emma E. George, Ben Knowles, Douglas S. Naliboff, Cameron A. Smurthwaite, Linda Wegley Kelly, Andreas F. Haas, Forest L. Rohwer

**Affiliations:** 1 Department of Biology, San Diego State University, San Diego, CA, United States of America; 2 Department of Marine Biology, Universidade Federal do Rio de Janeiro, Rio de Janeiro, Brazil

**Keywords:** Coral-algal interactions, Bioenergetics, Yield to power switch, Heterotrophic microbes, Microbialization, Ecosystem phase shifts

## Abstract

Human impacts are causing ecosystem phase shifts from coral- to algal-dominated reef systems on a global scale. As these ecosystems undergo transition, there is an increased incidence of coral-macroalgal interactions. Mounting evidence indicates that the outcome of these interaction events is, in part, governed by microbially mediated dynamics. The allocation of available energy through different trophic levels, including the microbial food web, determines the outcome of these interactions and ultimately shapes the benthic community structure. However, little is known about the underlying thermodynamic mechanisms involved in these trophic energy transfers. This study utilizes a novel combination of methods including calorimetry, flow cytometry, and optical oxygen measurements, to provide a bioenergetic analysis of coral-macroalgal interactions in a controlled aquarium setting. We demonstrate that the energetic demands of microbial communities at the coral-algal interaction interface are higher than in the communities associated with either of the macroorganisms alone. This was evident through higher microbial power output (energy use per unit time) and lower oxygen concentrations at interaction zones compared to areas distal from the interface. Increases in microbial power output and lower oxygen concentrations were significantly correlated with the ratio of heterotrophic to autotrophic microbes but not the total microbial abundance. These results suggest that coral-algal interfaces harbor higher proportions of heterotrophic microbes that are optimizing maximal power output, as opposed to yield. This yield to power shift offers a possible thermodynamic mechanism underlying the transition from coral- to algal-dominated reef ecosystems currently being observed worldwide. As changes in the power output of an ecosystem are a significant indicator of the current state of the system, this analysis provides a novel and insightful means to quantify microbial impacts on reef health.

## Introduction

Scleractinian corals and benthic algae harbor diverse and abundant microbial and viral communities (Herndl & Velimirov, 1986; Rohwer et al., 2001; Kellogg, 2004; Wegley et al., 2004; Marhaver, Edwards & Rohwer, 2008) that are largely species specific and distinct from the surrounding water column (Rohwer et al., 2002; Knowlton & Rohwer, 2003; Bourne & Munn, 2005; Ritchie, 2006; Marhaver, Edwards & Rohwer, 2008; Barott et al., 2011; Nelson et al., 2011; Morrow et al., 2012a; Hester et al., 2015; Frade et al., 2016). Both holobiont-associated and planktonic microbes play a major role in reef trophic dynamics (Hoogenboom et al., 2015; Tremblay et al., 2015) and the biogeochemical cycling of the surrounding environment (Selmer, Ferrier-Pages & Cellario, 1993; Lesser et al., 2004; Wegley et al., 2007; Siboni et al., 2008; Raina et al., 2009; Fiore et al., 2010; Haas et al., 2010; Haas et al., 2011). Benthic macro- and microorganisms have also been shown to change the availability of various organic and inorganic nutrients (Wild et al., 2004). For example, primary producers alter the oxygen availability (Wild et al., 2010; Wangpraseurt et al., 2012; Haas et al., 2013; Jorissen et al., 2016) through photosynthetic and respiratory metabolism which concomitantly influences pH (Smith et al., 2013) in their immediate surrounding. Furthermore, corals and algae produce both hydrophilic and hydrophobic organic compounds which can affect neighboring organisms by serving as energy sources or bioactive/inhibitory allelochemicals (Ritchie, 2006; Smith et al., 2006; Nissimov, Rosenberg & Munn, 2009; Morrow et al., 2011; Morrow et al., 2012b; Rasher et al., 2011).

Understanding the extent of microbial influences and how they differentially affect ecosystem function has become increasingly important during times of global shifts in benthic reef community structure, in which turf and fleshy macroalgae are becoming the dominant benthic macroorganisms covering the substratum of many formerly coral-dominated reef ecosystems on a worldwide scale (McCook, 1999; McCook, Jompa & Diaz-Pulido, 2001; Hughes et al., 2003; Hughes et al., 2007; Smith et al., 2016). The resulting benthic fragmentation yields a higher likelihood of coral-algal interaction events (Barott et al., 2012a). Although the course and outcome of these interactions will consequently determine the community structure of the reef environment, the mechanisms involved in ongoing interaction events are still not fully understood. Whether directly through diseases (Bourne et al., 2009) or microbial vectoring (Nugues et al., 2004; Vega Thurber et al., 2012), or indirectly through harmful secondary metabolites (Hay, 1996; Morrow et al., 2011) or microbe induced hypoxia (Smith et al., 2006; Barott et al., 2009; Barott et al., 2012b; Gregg et al., 2013; Haas et al., 2013), microbial involvement in these interaction events is significant.

Algal-derived exudates can cause high levels of coral mortality due to drawdown of dissolved oxygen concentrations by microbial growth on the labile sugars in these exudates (Smith et al., 2006; Barott et al., 2009). These hypoxic conditions compromise coral health more than algal health, impacting their ability to compete for the limited substrate in reef environments (Haas et al., 2014). Furthermore, algal dominated patches of reefs have been found to yield communities consisting of a greater proportion of heterotrophic microbes, which contain a higher number of virulence factors (Dinsdale et al., 2008; Kelly et al., 2012). The positive feedback loop resulting from these interactions is described as the Dissolved Organic Carbon (DOC), Disease, Algae, and Microbes (DDAM) model (Kline et al., 2006; Barott & Rohwer, 2012). DDAM posits that increases in the percent cover of benthic macroalgae causes enhanced release of bioavailable DOC, which yields a copiotrophic microbial community (Nelson et al., 2013). In turn, this change in the microbial community structure leads to coral mortality through various pathogenicity mechanisms. The increase in coral mortality frees up benthic space for more algae to inhabit, which yields a positive feedback loop, ultimately resulting in coral death and algal dominated reef systems. Moreover, microbial communities growing on algal exudates have higher and less effective carbon turnover rates, thereby increasing local oxygen consumption rates (Haas et al., 2011) and altering the allocation of energy toward microbial activity and away from higher trophic levels (McDole et al., 2012). While this implies a change in the overall microbial bioenergetics thermodynamics in these communities (Haas et al., 2016), the direct connection between microbial metabolism and thermodynamics has yet to be investigated.

Work in the field of thermal physics has demonstrated that as open systems approach a steady state they begin to minimize energy dissipation (Onsager, 1931a; Onsager, 1931b; Prigogine & Nicolis, 1971). Dissipation is one way to measure the energy that is converted to heat and “wasted” by any process. In biological systems, thermodynamic theory (Wicken, 1980; Weber et al., 1989; Salthe, 1998) predicts that as ecosystems approach a steady state, they should foster a community which serves to minimize the dissipation and thus maximize the *yield* (amount of work done per unit energy input (J/J)) of the system (Onsager, 1931a; Onsager, 1931b; Prigogine & Nicolis, 1971). In contrast, perturbed ecosystems undergoing a phase shift transition will theoretically harbor a community that maximizes the *power output* (the amount of energy used per unit time (J/s)) of the system (Odum, 2007). Systems running at maximum power do so by operating at less than maximal efficiency (Odum & Pinkerton, 1955; DeVos, 1992). Maximizing power output is typically accomplished by proceeding at efficiencies no greater than 50% of the maximum reversible efficiency (Fig. S1) (Odum & Pinkerton, 1955). Thus, changes in the power output of an ecosystem may be a significant indicator of the current state of the system (Fath, Patten & Choi, 2001; Nielsen & Jorgensen, 2013).

Here, we used a novel combination of methods to characterize how benthic macroorganism interactions affect the metabolisms and thermal power output of reef-associated microbes. A combination of microcalorimetry with planar oxygen optode imaging (Gregg et al., 2013; Haas et al., 2013), flow cytometry, and epifluorescence microscopy was used to generate a detailed bioenergetic analysis of microbial assemblages associated with coral-algal interactions. Microcalorimetry provides a thermodynamic assessment of metabolic heat dissipation by respective communities. By directly measuring the amount of energy dissipated as heat per unit time (i.e., thermal power output measured in J/s or W) we assessed both the rate and efficiency of the microbial community metabolisms. Planar oxygen optodes allowed for a fine scale 2-dimensional visualization of the metabolic effects on O_2_ concentrations in the surrounding water-column (Gregg et al., 2013), and flow cytometry permitted the partitioning of autotrophic and heterotrophic microbes in these communities (Zubkov et al., 2000). Epifluorescence microscopy allowed us to tie the values obtained through the previous methods to the total microbial abundance. The results of this study provide insight as to how energy flows through the various metabolisms of coral and algal microbiomes, thus unraveling a possible mechanism responsible for determining the outcomes of coral-algal interactions.

## Methods

### Experimental design and sampling

**Figure 1:**
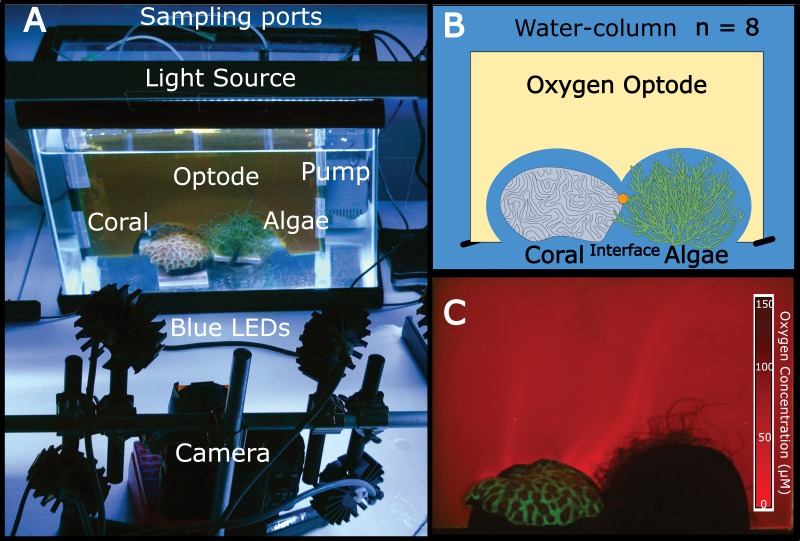
The experimental set-up. (A) Aquaria containing coral and algae were outfitted with a planar oxygen optode mounted vertically above the benthic macroorganisms. The light source provided 12 h dark-light cycles and the pump on the right side of the tank provided constant water flow. Optodes were excited with blue LED light and imaged using a Canon G11 camera. (B) Schematic of the experimental set-up (eight replicate aquariums were used). The orange dot represents the sampling location for the interface. (C) Representative image of a planar oxygen optode. Brighter red intensity indicates relatively lower oxygen concentrations.

Independent 18.9 L aquaria were filled with artificial seawater. One piece of coral (*Favia rotunda*) and one mass of algae (*Chaetomorpha crassa*) were placed in direct contact with one another in each tank at distances of >5 cm from aquaria walls (Figs. 1A and 1B). For each replicate (*n* = 8) a planar oxygen optode sheet (15 × 15 × 20 cm) was vertically fitted around the coral, algae, and interface (Fig. 1) to provide a two-dimensional colorimetric analysis of the O_2_ concentration (Fig. 1C). Polyethylene tubing (5 mm) connected to 21 gauge luer lock needles mounted to the back of the optode sheet served as sampling ports through which water was suctioned directly off the surface of the coral, the algae, and the interface between the two organisms via a 10 mL syringe. The aquaria were equilibrated for 36 h at 26–27 °C with a constant recirculating water flow at 75 L/hr provided by a submersible aquarium water pump (Aquatic Warehouse, San Diego, California, USA) with 12-hour dark/light cycles. Irradiance levels (150–180 µE/m^2^/s), temperature (26–27 °C), and flow rates (75 L/hr) were relatively similar to shallow tropical reef ecosystems. After preliminary equilibration, pictures were taken of the oxygen optodes, after which 5 mL of water was sampled from each of the three benthic areas (coral, algae, and interface) via the aforementioned sampling ports. Ambient water-column samples were also taken. This sampling scheme was conducted on eight separate biological replicates.

### Sample processing

Each sample was divided into aliquots for flow cytometry (1 mL), epifluorescence microscopy (1 mL), and calorimetry (3 mL). Flow cytometry samples were fixed with microscopy grade glutaraldehyde at 2% final concentration and then flash frozen in liquid N_2_ before being stored at −80 °C. Microscopy samples were fixed with microscopy grade paraformaldehyde at 1% final concentration, vacuum filtered onto a 0.02 µm Anodisc filter (Whatman inc., Florham Park, NJ, USA), stained with SYBR Gold (5 X final concentration; Invitrogen, Carlsbad, CA, USA), and mounted on microscope slides. Calorimetry samples were weighed and placed in a TAM III isothermal heat conduction microcalorimeter (TA Instuments, New Castle, DE, USA) for 8 h.

### Sample analyses

Flow cytometry samples were thawed at 37 °C and pipetted into a flat bottomed 96-well-plate on ice. Analysis was performed with a BD FACS-Canto via the high-throughput sampler unit for enumeration utilizing the methods of Zubkov et al. (2000). For enumeration of autotrophic microbe populations, 100 µL of sample were collected for analysis on standard mode using the 488 nm excitation laser. Bivariate plots were used to analyze the sample for chlorophyll fluorescence (red), which were done in the PerCP-Cy5-5 channel (670 nm long pass filter preceded by a 655 nm long pass mirror) and the phycoerytherin (PE) channel (585/42 band pass filter preceded by a 556 nm long pass mirror). For autotrophic populations, threshold gating was determined by using 0.02 µm filtered seawater. Yellow-green fluorescent microsphere beads (0.75 µm) were used to control for sample volume analyzed. To control for consistency between plates and daily runs, a standard seawater sample collected from San Diego, California was used. This control was used for both autotrophic and total microbe enumerations. One hundred µL sample volume was collected and analyzed for SYBR fluorescence, which was excited by the 488 nm laser and detected in the FITC channel (530/30 nm band pass filter preceded by a 502 nm long pass mirror). Threshold gating for the heterotrophic populations was determined by using unstained representative coral reef water, and to verify the amount of background associated with the instrument. Data was collected on FACSDiva 6.1.1 and analyzed using FlowJo 7.6.5. In order to enumerate heterotrophic microbe populations, the comparative autotrophic populations were subtracted from the total microbial counts.

The oxygen optode sheets were prepared and analyzed following (Haas et al., 2013). Prior to each experiment oxygen optodes were calibrated under identical temperatures (26 °C) with known oxygen concentrations. For this calibration, filtered seawater was dosed with nitrogen gas to obtain eight different concentrations of oxygen ranging from 100% air-saturation to anoxia. Images were taken approximately every 30 µM step until the seawater was anoxic. The resulting oxygen concentrations were constantly measured for comparison using an LBOD101 luminescent oxygen probe.

During experiments, oxygen optodes were imaged using a G11 (Cannon, USA) camera placed at ∼25 cm from the aquarium. All images were captured in RAW format with identical settings of ISO 200, f ∖8 and shutter speed of 1.3 s. Four Rebel Royal Blue light emitting diodes (LED) with a λ-peak of 445 nm (Philips-Luxeon; Philips Lighting, Toronto, Canada) were used as the excitation source in combination with a 470 nm short pass filter (UQG Optics, UK). To prevent the excitation source from contaminating the luminescent signal, a Schott 530 nm long pass filter (UQG Optics, Cambridge, UK) was mounted on the camera lens. All images were taken in the absence of ambient light.

Absolute O_2_ concentrations were determined by measuring the red pixel intensity (oxygen-dependent dye) to green pixel intensity (oxygen-independent antenna dye) using the methods of Haas et al. (2013). Briefly, each RAW file was converted into two16-bit TIFF images (i.e., red and averaged green) (RawHide v0.88.001; My-Spot Software, USA). The pixel information from the red and green channel images were imported into MATLAB and further analyzed using the image toolbox. The red and green intensity values were obtained for each pixel and used to calculate the pixel intensity ration.

Isothermal calorimetry was conducted using a TAM III multi-channel isothermal heat-conduction microcalorimeter. Samples were placed in the machine within an hour of the sampling process. Samples were lowered into the measurement position after a 30 min equilibration period. Measurements on temperature (K), cumulative heat (J), and instantaneous heat flow (W) were taken continuously for each channel for 8 h at 299 K. Heat and heat flow were subsequently normalized by the total mass (g) of the respective sample.

### Statistical analyses

All tests were conducted with an alpha of 0.05 (95% confidence level). A one-way analysis of variance (ANOVA) followed by a Student’s T-test *post hoc* analysis were used to test for significant differences in microbial power outputs (µW), microbial abundances (cells per mL ×10^6^), heterotroph to autotroph ratios, and dissolved oxygen (µM) by treatments (i.e., an effect of coral, algae, interface). The data was further analyzed with linear regression by treatments over time compared to power output, normalized heat (J/g) compared to heterotroph to autotroph ratio, and normalized heat (J/g) to microbial abundance (cells per mL ×10^6^). To determine if there was a significant effect of treatment area on the heat production over time, we used and ANCOVA test for the analysis of covariance of power output between treatment groups over time. All statistical analyses were performed using JMP 10 software (SAS Software). Our statistical results are listed below and in Tables  S1–S4.

## Results

### Macroorganism-associated microbial thermodynamics

**Figure 2:**
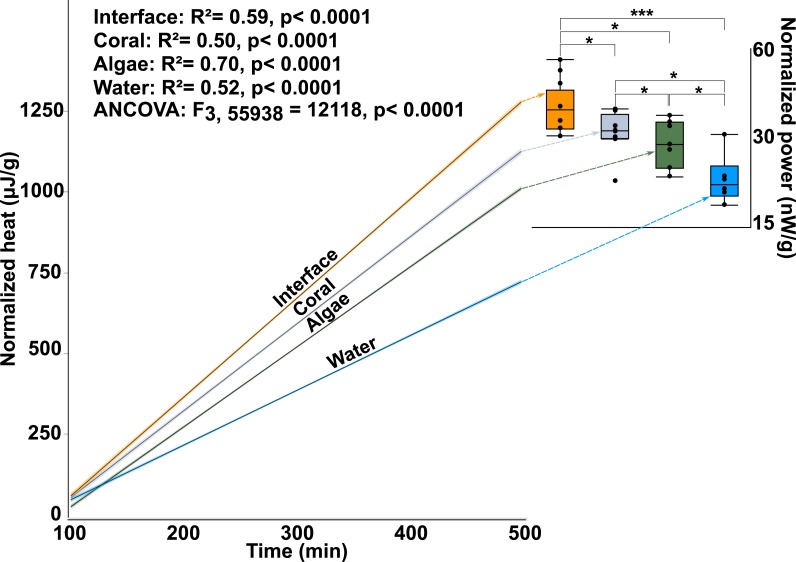
Microbial power output. The *y*-axis is the total heat (µJ, microjoules per g, gram seawater). The *x*-axis is time in minutes, min. The lines represent the best fit for the mean of the eight replicates. The inset shows total power output of microbial communities (nW/g, nanowatts per gram). The power output represents the first derivative of the lines in the main figure. (^∗∗∗^ *T*-test, *p* ≤ 0.001 ^∗^ *T*-test, *p* ≤ 0.05).

Calorimetry demonstrated that microbial thermodynamic power output (interface: 33.652 ± 1.65600B5 J; coral: 28.552 ± 1.65600B5 J; algae: 26.534 ± 1.77000B5 J; water-column: 19.4359 ± 1.91200B5 J; mean ± s.e.m. *n* = 8) was significantly different between the four treatments (One-way ANOVA: *F*_3,28_ = 10.7653*p* < 0.0001) with the interface-associated microbial community having a significantly higher power output than either the coral- or algae-associated communities alone (Fig. 2 inset; *Post hoc t*-test: interface-coral *p* = 0.0390, interface-algae *p* = 0.0070) (Table S1). Furthermore, both coral- and algae-associated microbial communities had significantly higher power output than the microbial communities in the surrounding water (Fig. 2 inset) (*Post hoc t*-test: coral-water *p* = 0.0014, algae-water *p* = 0.0116) and in the 0.02 µm filtered seawater control (*Post hoc t*-test: *p* < 0.0001). Integration of the total heat production over time (Fig. 2 and inset) demonstrates a significant effect of the sampling area on the heat production over time (ANOCOVA: *F*_3,55938_ = 12, 118 *p* < 0.0001). The interface community not only produces significantly more total heat over the eight hours in the calorimeter, but also has a significantly higher rate (5.13 ± 0.00347 µJ/s) of heat production (i.e., power) at all points in time (Fig. 2 inset) than the microbial communities associated with coral (4.50 ± 0.003702 µJ/s) or algae (4.14 ± 0.002416 µJ/s; mean ± s.e.m.).

This data demonstrates that there are small-scale spatial differences in the way that energy flows though macroorganism-associated microbial communities. More specifically, the microbial assemblages associated with the coral-algal interface have a higher thermodynamic power output than the microbial communities associated with either macroorganism alone. The higher microbial power output at the interface indicates that the community metabolism at the interface was faster and less efficient, as it used more energy per unit time, and dissipated more of that energy as heat.

### Microbial community trophic structure

**Figure 3:**
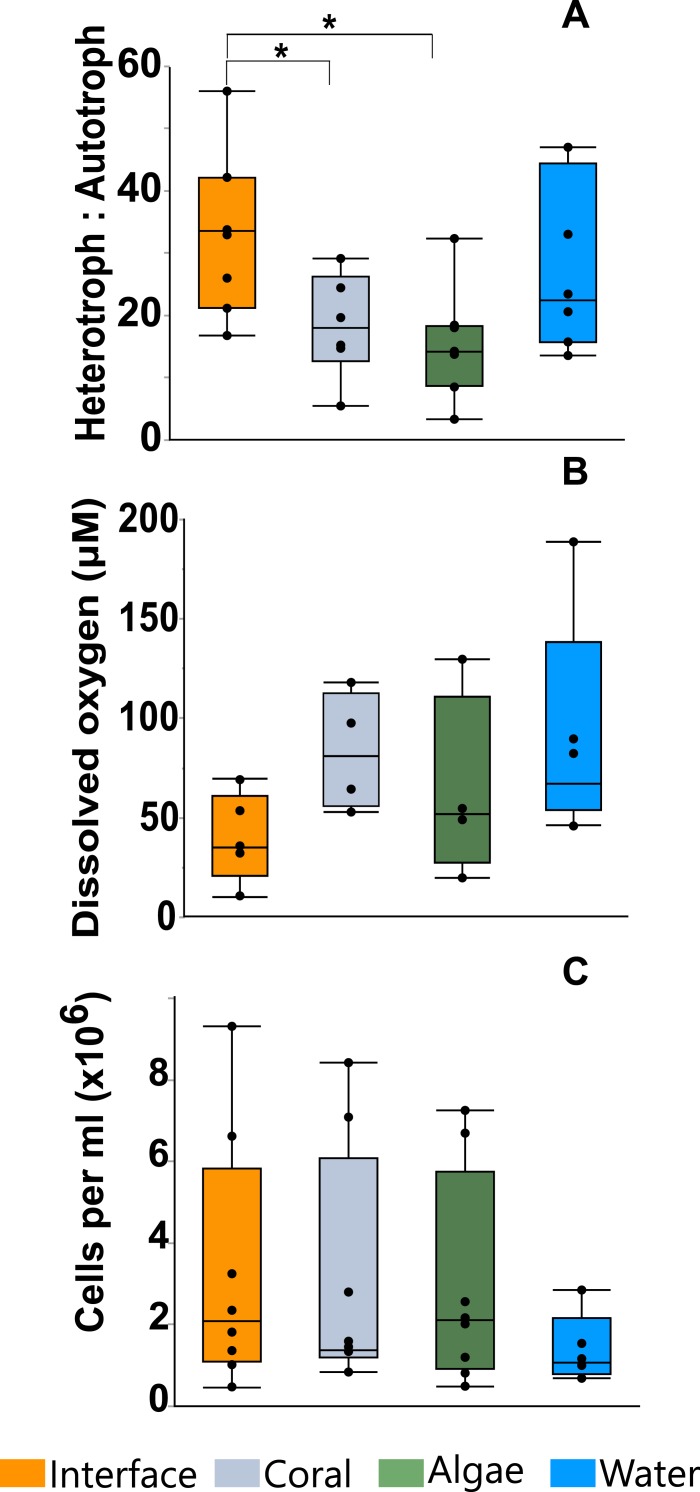
Microbial assessment at the coral-algal interface. (A) Heterotroph to autotroph ratio. (B) Dissolved oxygen concentration (micromolar, µM). (C) Total microbial abundance (cells per milliliter, ml) (* *T*-test *p* ≤ 0.05).

**Figure 4:**
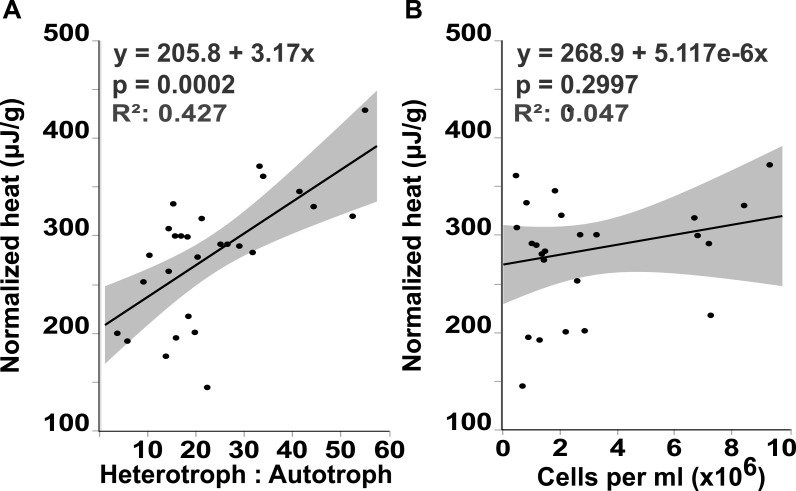
Heat output linear regression analysis. Total heat output of the microbial community (µJ, microjoules) plotted against (A) the heterotroph to autotroph ratio and (B) the microbial abundance (cells per milliliter, ml)

Flow cytometry analysis revealed that the interface microbial community was significantly more heterotrophic (32.946 ± 4.3231) than the communities associated with coral (18.4932 ± 4.6695) or algae (15.6103 ± 4.3231; mean hetrerotroph: autotroph ratio ± s.e.m.) (Fig. 3A) (One-way ANOVA: *F*_3,26_ = 3.4106 *p* = 0.0345, *Post hoc T*-test: interface-coral *p* = 0.0328, interface-algae *p* = 0.0094) (Table S2). This shift in community metabolism to a more heterotrophic microbial consortium at the interface correlates significantly with the total heat output of the microbial communities (Fig. 4A) (*R*^2^:0.427; ANOVA, *F*_1,26_ = 18.6651 *p* = 0.0002). Whereas, total microbial abundance was not significantly different between sampling sites (Fig. 3C and Table S4; one-way ANOVA: *F*_3,26_ = 0.5631 *p* = 0.6444) (Table S3), and was not a strong predictor of heat production (Fig. 4B) (*R*^2^:0.047; ANOVA, *F*_1,27_ = 1.7697 *p* = 0.2997), indicating that the shift towards a more heterotrophic community metabolism is responsible for the increased power at the coral-algal-interaction interface.

### Biological oxygen demand

Planar oxygen optodes (Gregg et al., 2013) provided a fine scale 2-dimensional assessment of the O_2_ concentrations associated with the coral-algal interactions. Figure 1C presents a representative picture of the oxygen concentrations visualized in a typical coral-algal interaction experiment. Two dimensional oxygen concentration assessment demonstrates a general trend of an O_2_ decline at the coral-algal interface relative to the oxygen levels associated with coral, algae, and the watercolumn (Figs. 1C and 3B; O_2_ concentrations: interface: 39.8299 ± 18.7 µM ; coral: 82.422 ±  20.907 µM; algal: 63.4961 ± 20.91; water-column: 90.3468 ± 18.7µM; mean ± s.e.m.) (Table S4), in accordance with previous observations by Haas et al. (2013). While lowered O_2_ concentrations at the interface were not statistically significant, they are likely biologically significant, as the interface is the only area in which O_2_ concentrations were significantly below the 70 µM O_2_ concentration at which >50% of coastal marine organisms die from hypoxia (Vaquer-Sunyer & Duarte, 2008).

## Discussion

Our results demonstrate a shift in community metabolism towards heterotrophic dominance at the coral-algal interaction interface (Fig. 3A). This leads to an increase in catabolic reactions, which use biologically available oxygen; in turn, lowering O_2_ concentration at the interface between the macroorganisms (Figs. 3B and 1C). The unique microbial community metabolism at these interfaces (Fig. 3A) yields an increase in thermodynamic power (Fig. 2 and 4A). There are a number of *in situ* measurements that provide phenomenological support that there is a bioenergetic shift of the microbial community at the interface (e.g., Barott et al., 2009; Barott et al., 2012a; Wangpraseurt et al., 2012; Jorissen et al., 2016) However, there is no direct experimental evidence connecting these changes in community metabolism to the underlying thermal physics. Although it would be best to make these measurements *in situ*, there is currently no technology available to make thermodynamic measurements at this resolution on the reef itself. Thus, we provide experimental tests for this previously unconsidered mechanism underlying coral reef phase transitions using microcosm coral-algal interactions.

### Nonequilibrium thermodynamics and ecology

This study demonstrated that power output at the coral-algal interaction interface is significantly higher than in the communities associated with either of the single organisms. This was evident through higher microbial heat production rates (Fig. 2) at interfaces compared to areas distal from them. These measurements support prior observations that coral-algae interfaces foster microbial communities and metabolic profiles unique from those associated with either coral or algae (Barott et al., 2012c; Haas et al., 2013). This study further identifies small-scale, spatial alterations in the thermodynamics of microbial communities associated with specific areas across the coral-algal interface. Specifically, the data confirms a *yield-to-power switch* (as suggested by Haas et al., 2016) occurring at the interface between coral and algae. A better understanding of the thermodynamics of coral-algal interactions may provide bioenergetic data that can be used for modeling reef ecosystems to predict the systems’ trajectories and possibly devise plans for ecological remediation.

### Bioenergetic mechanisms of the DDAM feedback model

At the coral-algal interface, algae exude Dissolved Organic Carbon (DOC) in close proximity to coral, which causes a shift in the microbial community towards more heterotrophic metabolisms (Fig. 3A). As heterotrophic microbial metabolism is favored by these interaction events, the microbial community metabolism is shifted towards more copiotrophic consumers relative to the amount of autotrophic producers (Fig. 3A). This affects coral reef systems in two ways. First, copiotrophs have greater net oxygen consumption, which causes localized hypoxia at the coral-algal interface (Figs. 1C and 3B), (Barott et al., 2012b; Wangpraseurt et al., 2012; Haas et al., 2013). Second, this microbial community shift towards more heterotrophic consumers means that a greater portion of the available energy will be utilized by the microbial fraction of the ecosystem leaving less energy for higher trophic levels, a process referred to as microbialization (McDole et al., 2012; Haas et al., 2016).

**Figure 5:**
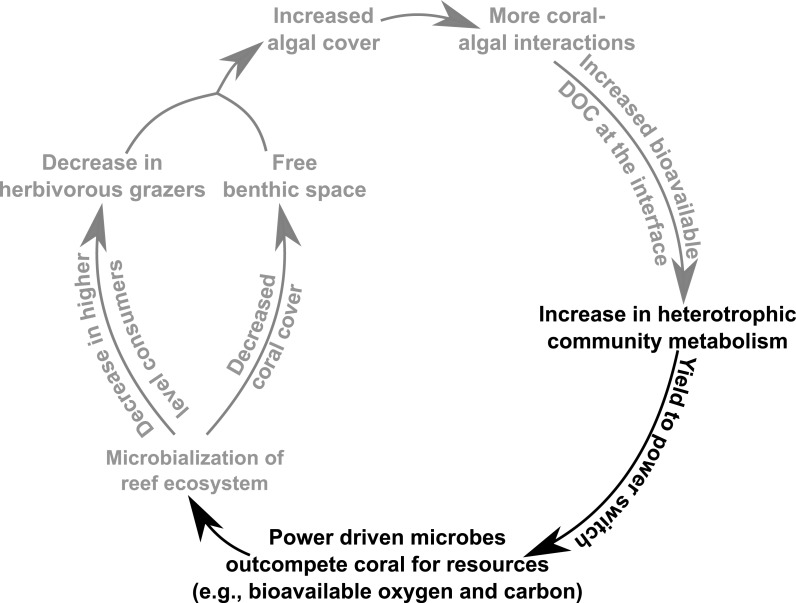
Conceptual depiction of the DDAM feedback model. Conceptual depiction of the DDAM feedback model with known linkages shown in grey. Black text indicates the bioenergetic and thermodynamic mechanisms associated with the DDAM loop established by this study.

Microbialization is a metric of the proportion of energy allocated to the microbial fraction of an ecosystem (i.e., a measure of the trophic cascading from microbes to macro-organisms) (McDole et al., 2012). One cause of microbialization on coral reefs is over-fishing (Jackson et al., 2001), which reduces grazing pressure on algae. Releasing macroalgae from grazing begins the DDAM positive feedback loop where DOC released by the un-grazed algae enriches for a copiotrophic, bacterial community, ultimately leading to coral mortality and more benthic space for algae. Our data suggests that a possible underlying mechanism in the DDAM loop (Kline et al., 2006; Barott & Rohwer, 2012) and the subsequent microbialization of coral reef ecosystems is the higher power output of the microbial communities at the coral-algal interaction interface. The increase in power—that is, energy used per unit time—at the interaction interface (Fig. 2) stems from an augmentation in net heterotrophy at the interface (Figs. 3A and 4A). This switch to a microbial community with higher thermodynamic power coupled to the increase in heterotrophic metabolisms, such as oxidative respiration, creates localized areas of hypoxia (Figs. 1C and 3B), which damages the coral tissue, ultimately leading to coral mortality (Fig. 5).

### Micro-scale and whole-reef-scale dynamics

Field studies have shown that increased macroalgal cover is significantly correlated with a net increase in heterotrophic microbial communities (Dinsdale et al., 2008; Kelly et al., 2012) and a shift toward faster, lower yield metabolisms (Haas et al., 2016). Our data suggest that the increase in power and net heterotrophy observed on a reef-wide-scale could be due to the small-scale (on the order of 10 µm–1 mm) spatial dynamics occurring at the coral-algal interface. That is, the altered community metabolisms and bioenergetics could possibly stem from the increased number of coral-algal interactions that occur as algae begins to increase in benthic abundance. This raises an interesting question: to what extent are the activities observed at the coral-algal interface driving the large-scale bioenergetic dynamics observed on the reef as a whole? Future work should focus on understanding the ways in which the small-scale activities at the interface affect whole reef dynamics. *In silico* modeling may provide a better understanding of how small-scale interaction events affect the overall bioenergetics of reef ecosystems.

## Conclusions

The findings presented here suggest that as coral reef ecosystems are perturbed by algal derived DOC, there is a shift in the microbial system from a community optimized for efficiency to one that is optimized to perform at maximum power. This yield-to-power switch of the microbial community allows the microbes to outcompete corals for bio-available oxygen, thus providing a competitive advantage to surrounding macroalgae. In this way, the yield-to-power switch is posited to be an underlying energetic mechanism involved in the microbialization of coral reefs and the DDAM positive feedback loop (Fig. 5), with potentially profound ecosystem impacts. As this change in microbial mediated reef energetics may be the mechanism leading to the ongoing algal dominations of benthic areas that were once inhabited by coral, an understanding of coral reef microbial bioenergetics serves as an indicator of coral reef health and provide important insight to predict the future trajectory of these valuable ecosystems.

##  Supplemental Information

10.7717/peerj.3423/supp-1 Figure S1 Conceptual depiction of power versus yield Power -the measure of energy flow per unit time (Joules/ second)—is maximized at 50% yield—the dimensionless measure of energetic output per unit energy input (Joules/ Joules), also referred to as efficiency. Figure adapted and modified from Odum & Pinkerton (1955).

10.7717/peerj.3423/supp-2 Table S1 Statistical output of one-way ANOVA and subsequent Student *t*-test *post hoc* analysis for power output (µW) normalized by weight of the sample (g)

10.7717/peerj.3423/supp-3 Table S2 Statistical output of one-way ANOVA and subsequent Student *t*-test *post hoc* analysi *s* for heterotroph: autotroph ratios

10.7717/peerj.3423/supp-4 Table S3 Statistical output of one-way ANOVA and subsequent Student *t*-test *post hoc* analysi*s* for dissolved oxygen concentration (µM)

10.7717/peerj.3423/supp-5 Table S4 Statistical output of one-way ANOVA and subsequent Student *t*-test *post hoc* analysi*s* for total cellular abundance (cells/ mL)

10.7717/peerj.3423/supp-6 Data S1 Calorimetry raw data Raw data exported from the TAM III calorimeter for the first experimental replicate.

10.7717/peerj.3423/supp-7 Data S2 Calorimetry raw data Raw data exported from the TAM III calorimeter for the sixth experimental replicate.

10.7717/peerj.3423/supp-8 Data S3 Calorimetry raw data Raw data exported from the TAM III calorimeter for the seventh experimental replicate.

10.7717/peerj.3423/supp-9 Data S4 Calorimetry raw data Raw data exported from the TAM III calorimeter for the fourth experimental replicate.

10.7717/peerj.3423/supp-10 Data S5 Calorimetry raw data Raw data exported from the TAM III calorimeter for the third experimental replicate.

10.7717/peerj.3423/supp-11 Data S6 Calorimetry raw data Raw data exported from the TAM III calorimeter for the eighth experimental replicate.

10.7717/peerj.3423/supp-12 Data S7 Calorimetry raw data Raw data exported from the TAM III calorimeter for the fifth experimental replicate.

10.7717/peerj.3423/supp-13 Data S8 Calorimetry raw data Raw data exported from the TAM III calorimeter for the second experimental replicate.

10.7717/peerj.3423/supp-14 Data S9 Oxygen optode raw data Oxygen optode raw data from the 1 replicate.

10.7717/peerj.3423/supp-15 Data S10 Oxygen optode raw data Oxygen optode raw data from the second replicate.

10.7717/peerj.3423/supp-16 Data S11 Oxygen optode raw data Oxygen optode raw data from the fourth replicate.

10.7717/peerj.3423/supp-17 Data S12 Oxygen optode raw data Oxygen optode raw data from the third replicate.

10.7717/peerj.3423/supp-18 Data S13 Flow cytometry raw data Raw data of unstained samples exported from the flow cytometer BD FACS-Canto and analyzed in Figs. 3 and 4.

10.7717/peerj.3423/supp-19 Data S14 Flow cytometry raw data Raw data of unstained samples exported from the flow cytometer BD FACS-Canto and analyzed in Figs. 3 and 4.

10.7717/peerj.3423/supp-20 Data S15 Flow cytometry raw data Raw data of unstained samples exported from the flow cytometer BD FACS-Canto and analyzed in Figs. 3 and 4.

10.7717/peerj.3423/supp-21 Data S16 Flow cytometry raw data Raw data of stained samples exported from the flow cytometer BD FACS-Canto and analyzed in Figs. 3 and 4.

10.7717/peerj.3423/supp-22 Data S17 Flow cytometry raw data Raw data of stained samples exported from the flow cytometer BD FACS-Canto and analyzed in Figs. 3 and 4.

10.7717/peerj.3423/supp-23 Data S18 Flow cytometry raw data Raw data of stained samples exported from the flow cytometer BD FACS-Canto and analyzed in Figs. 3 and 4.

## Additional Information and Declarations

### Data Availability

The following information was supplied regarding data availability: The raw data has been supplied as Supplementary Files.
