# Supplementary figures and images for: Microbial bioenergetics of coral-algal interactions
Source: PeerJ. 2017 Jun 21;5:e3423. doi: 10.7717/peerj.3423 (PMC5482263; doi:10.7717/peerj.3423)

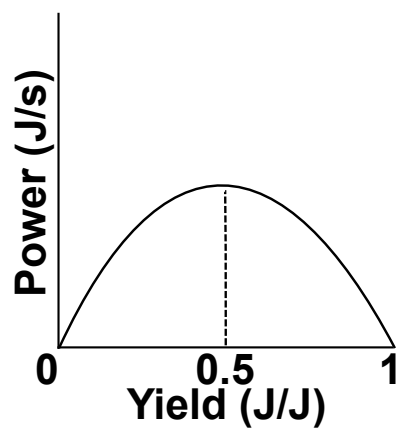

Supplement: Figure S1 — Power -the measure of energy flow per unit time (Joules/ second)—is maximized at 50% yield—the dimensionless measure of energetic output per unit energy input (Joules/ Joules), also referred to as efficiency. Figure adapted and modified from Odum & Pinkerton (1955). [file peerj-05-3423-s001.pdf]

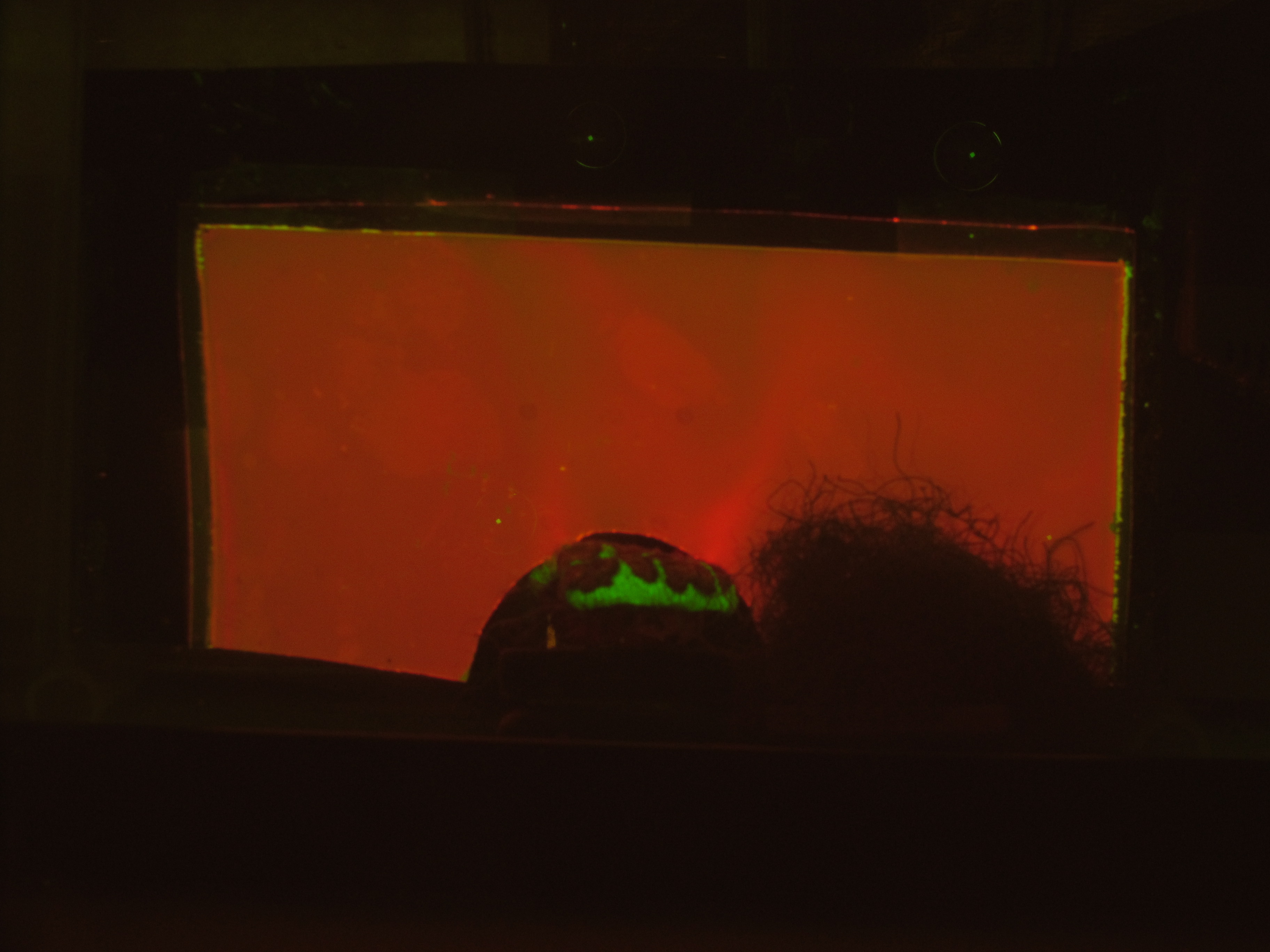

Supplement: Data S9 — Oxygen optode raw data from the 1 replicate. [file peerj-05-3423-s014.jpg]

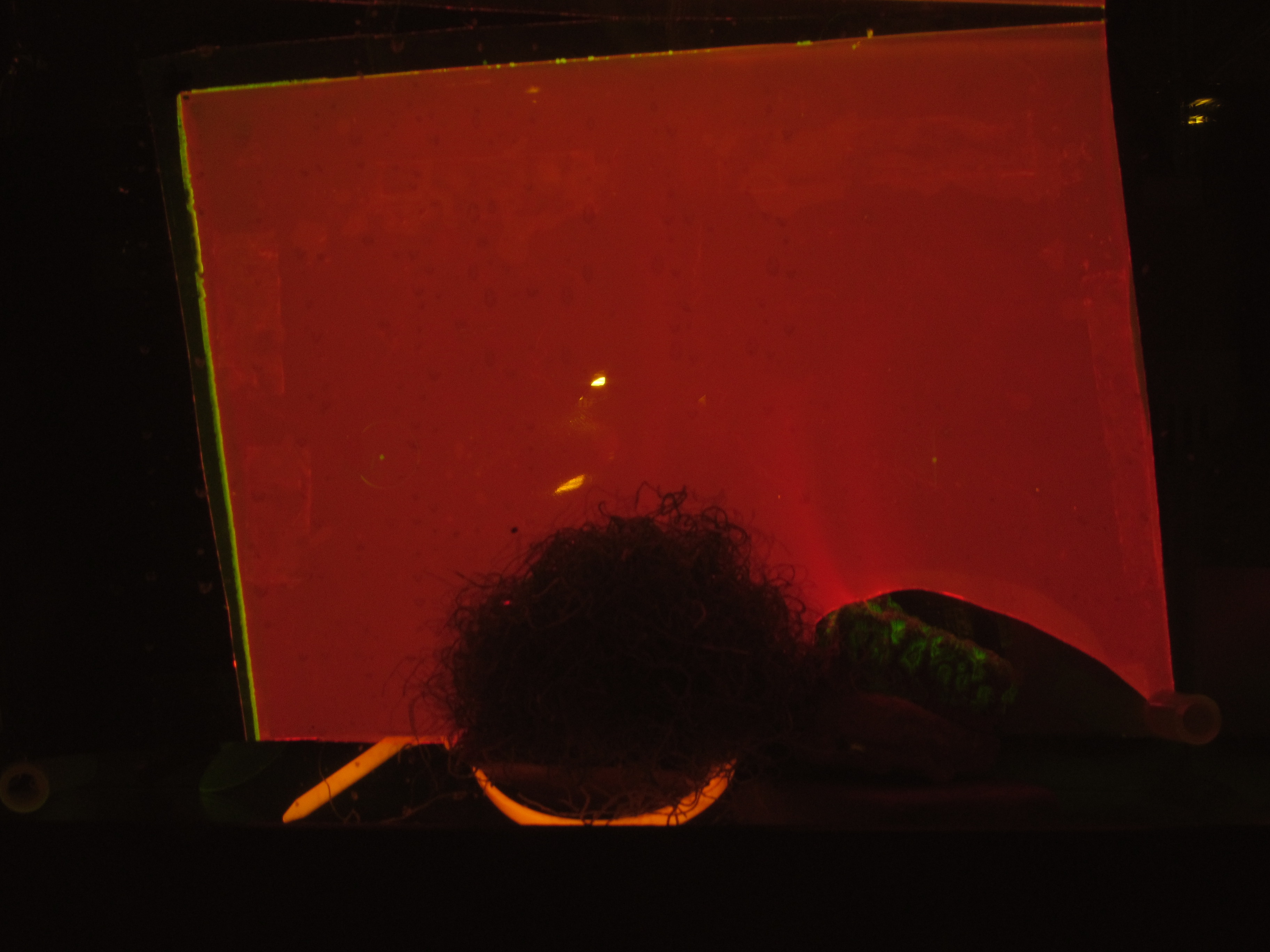

Supplement: Data S10 — Oxygen optode raw data from the second replicate. [file peerj-05-3423-s015.jpg]

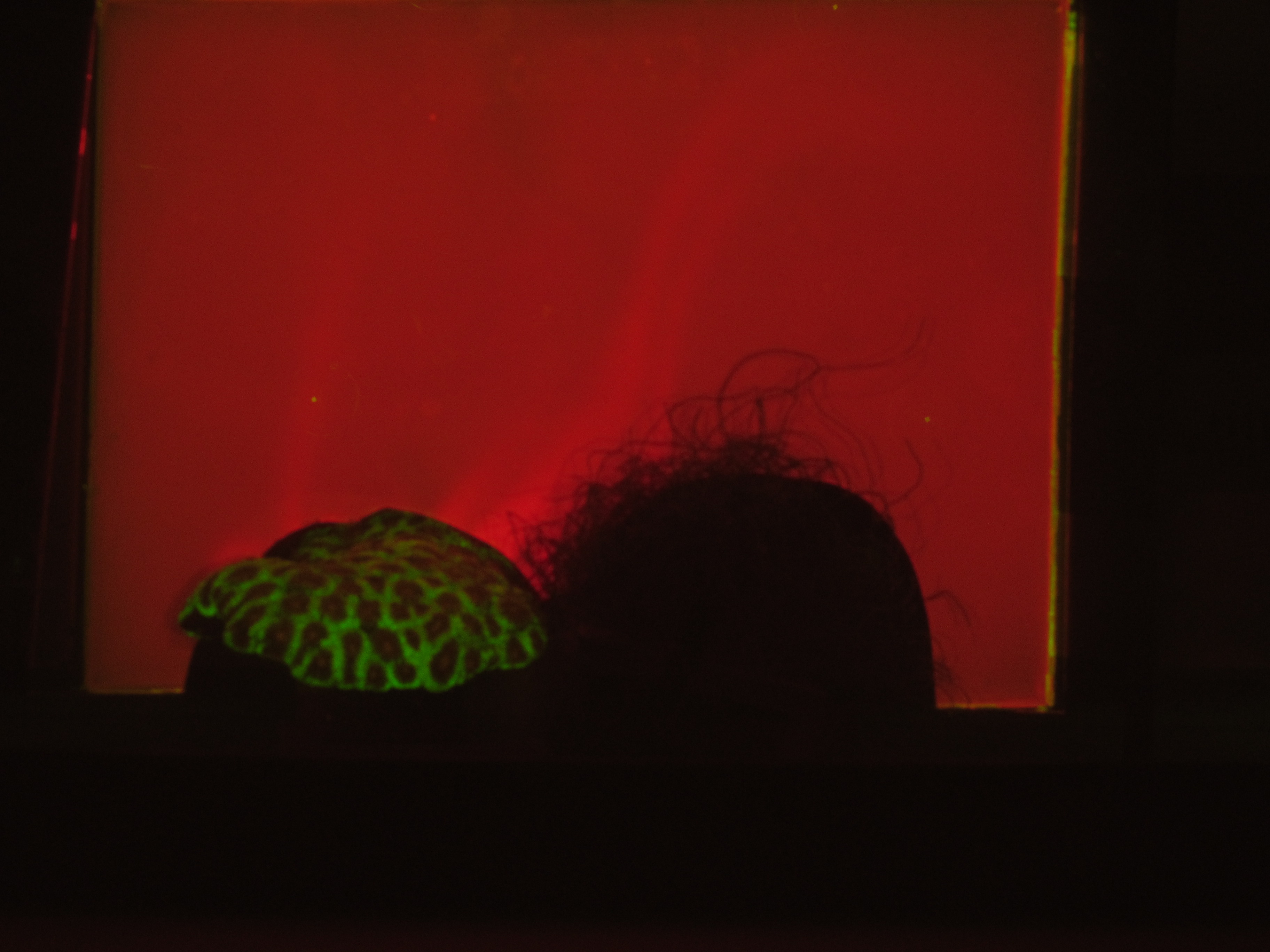

Supplement: Data S11 — Oxygen optode raw data from the fourth replicate. [file peerj-05-3423-s016.jpg]

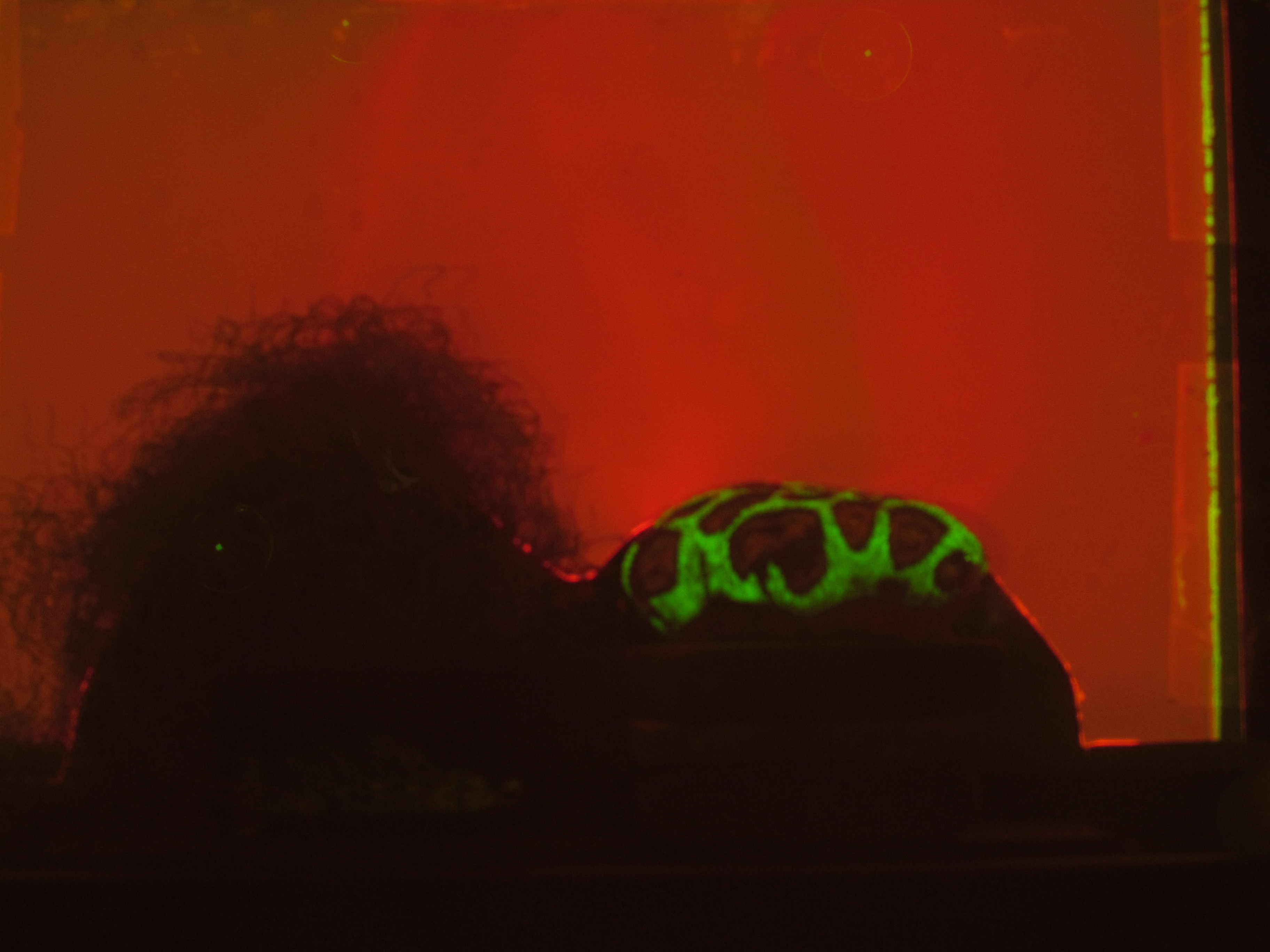

Supplement: Data S12 — Oxygen optode raw data from the third replicate. [file peerj-05-3423-s017.jpg]

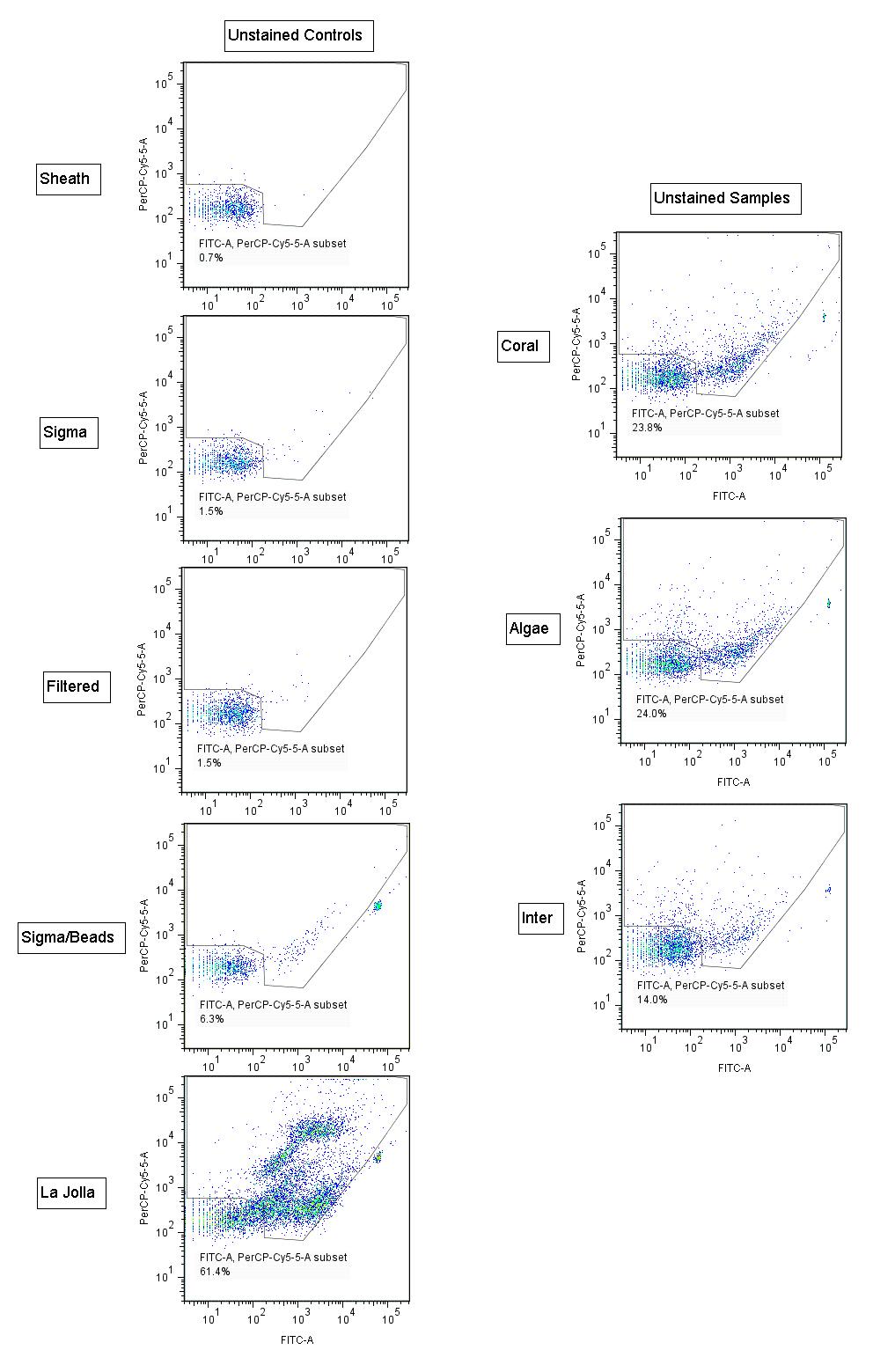

Supplement: Data S13 — Raw data of unstained samples exported from the flow cytometer BD FACS-Canto and analyzed in Figs. 3 and 4. [file peerj-05-3423-s018.jpg]

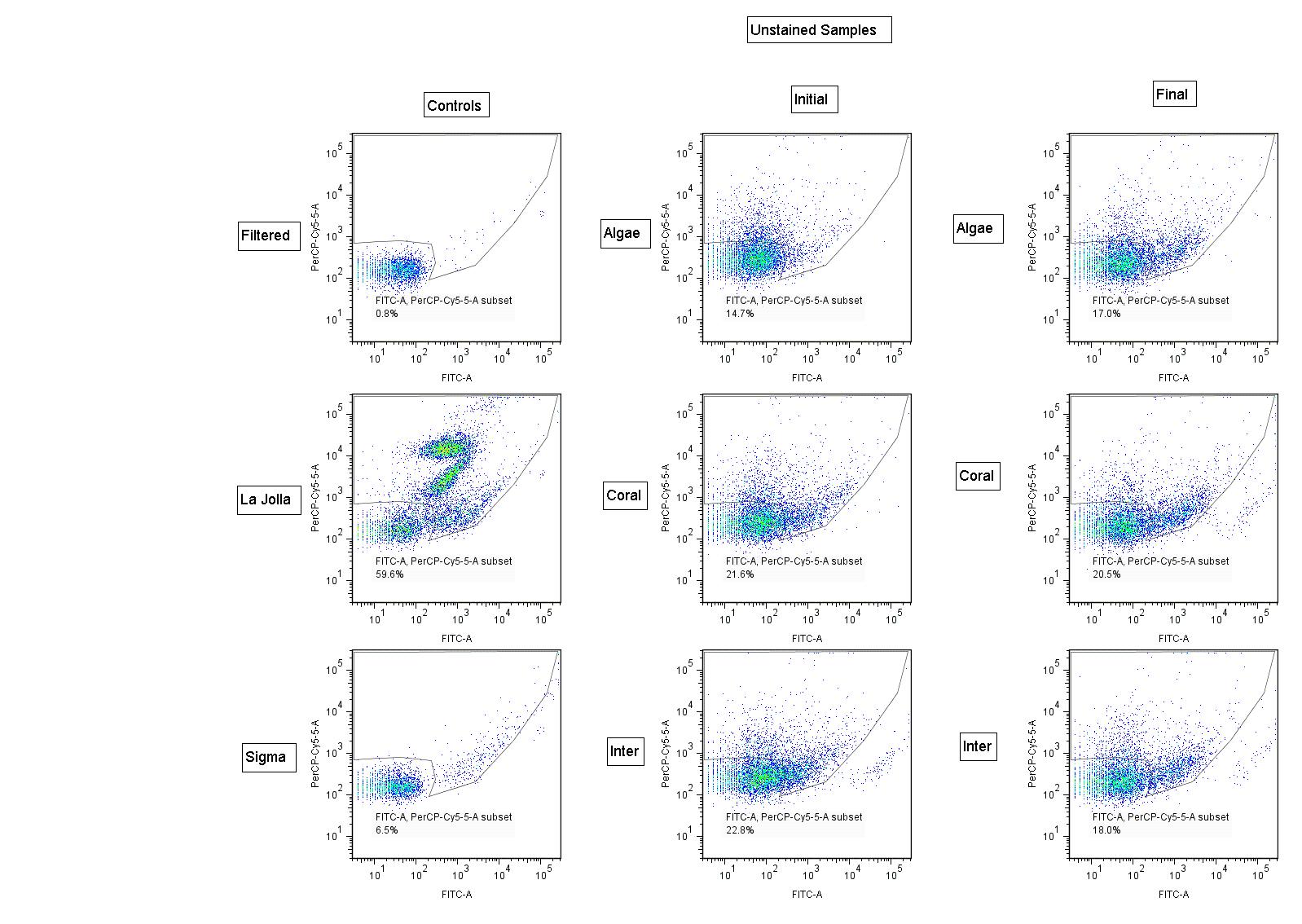

Supplement: Data S14 — Raw data of unstained samples exported from the flow cytometer BD FACS-Canto and analyzed in Figs. 3 and 4. [file peerj-05-3423-s019.jpg]

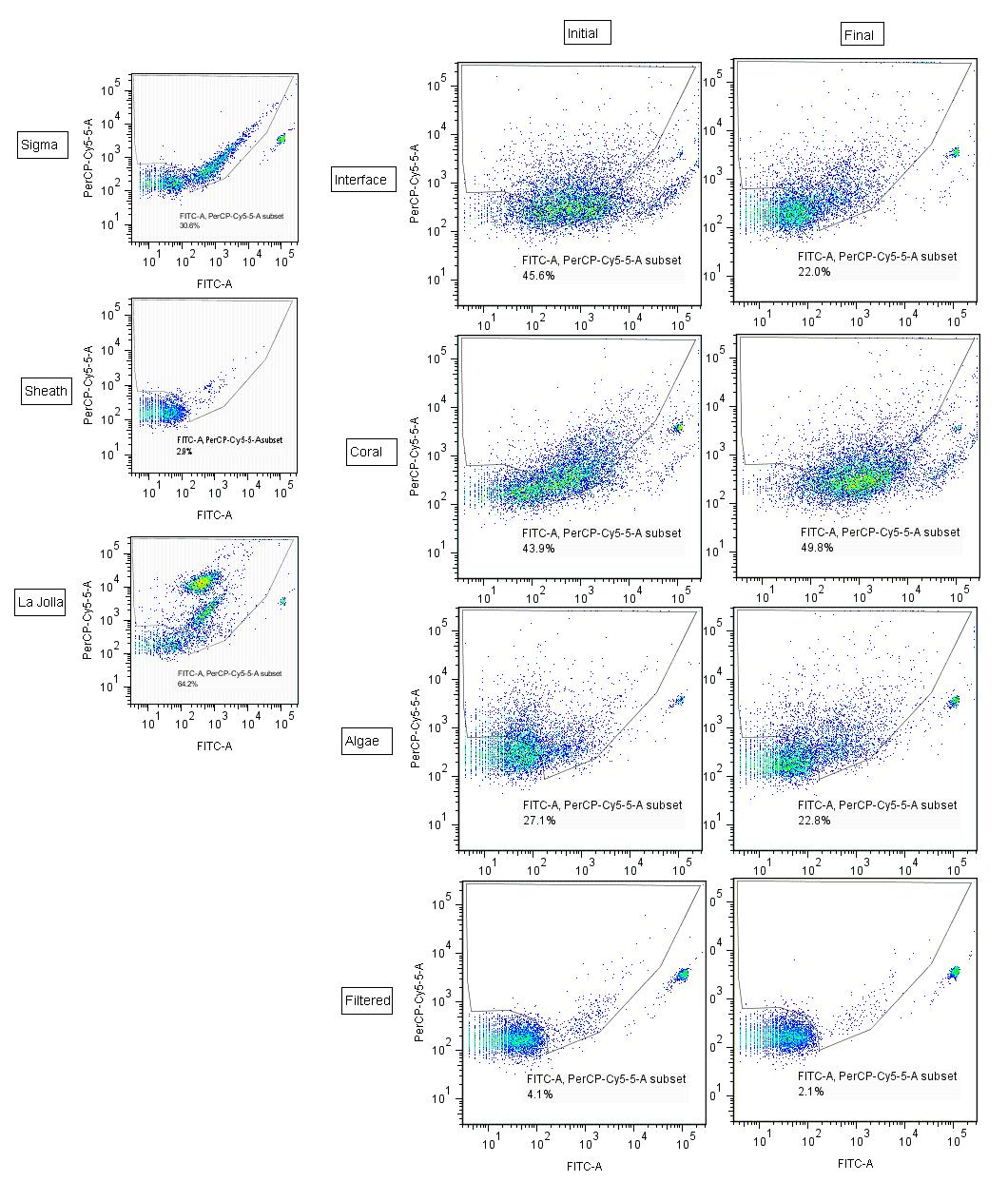

Supplement: Data S15 — Raw data of unstained samples exported from the flow cytometer BD FACS-Canto and analyzed in Figs. 3 and 4. [file peerj-05-3423-s020.jpg]

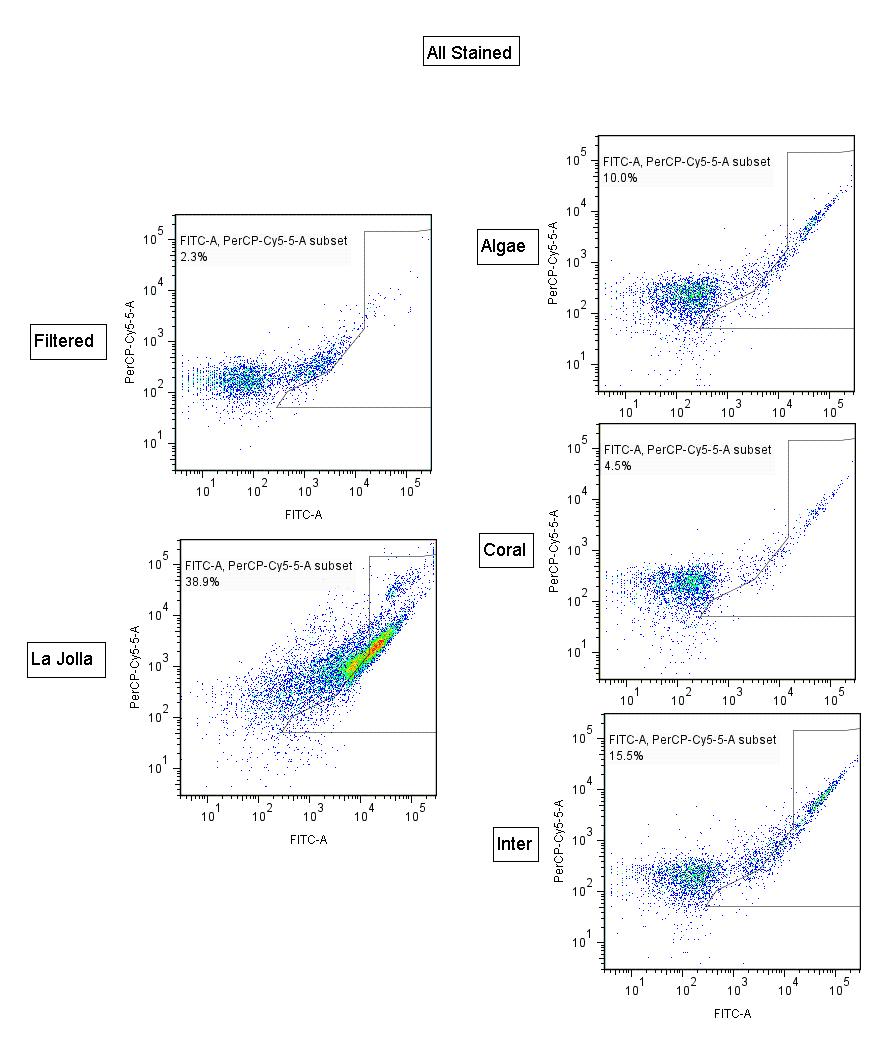

Supplement: Data S16 — Raw data of stained samples exported from the flow cytometer BD FACS-Canto and analyzed in Figs. 3 and 4. [file peerj-05-3423-s021.jpg]

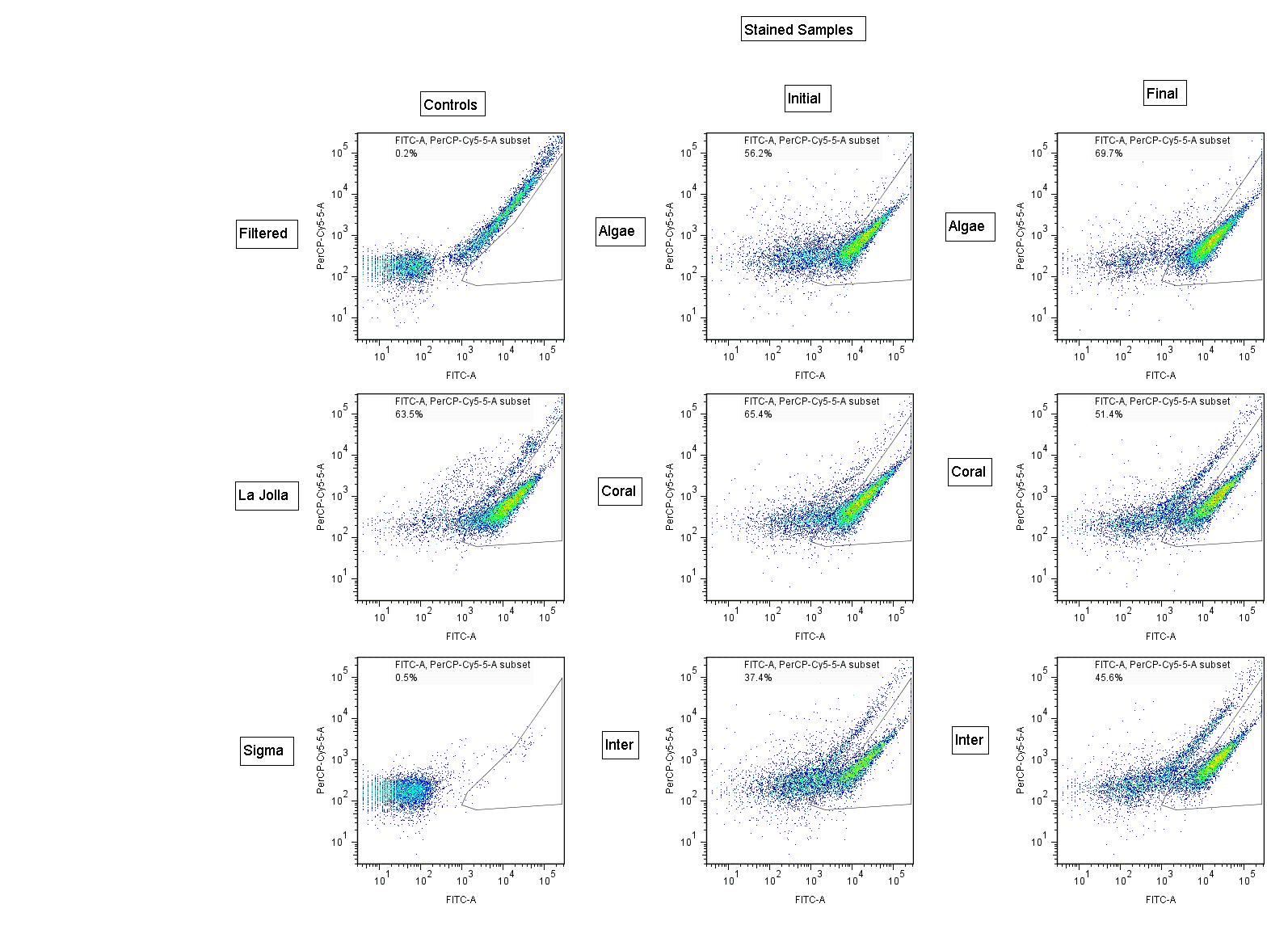

Supplement: Data S17 — Raw data of stained samples exported from the flow cytometer BD FACS-Canto and analyzed in Figs. 3 and 4. [file peerj-05-3423-s022.jpg]

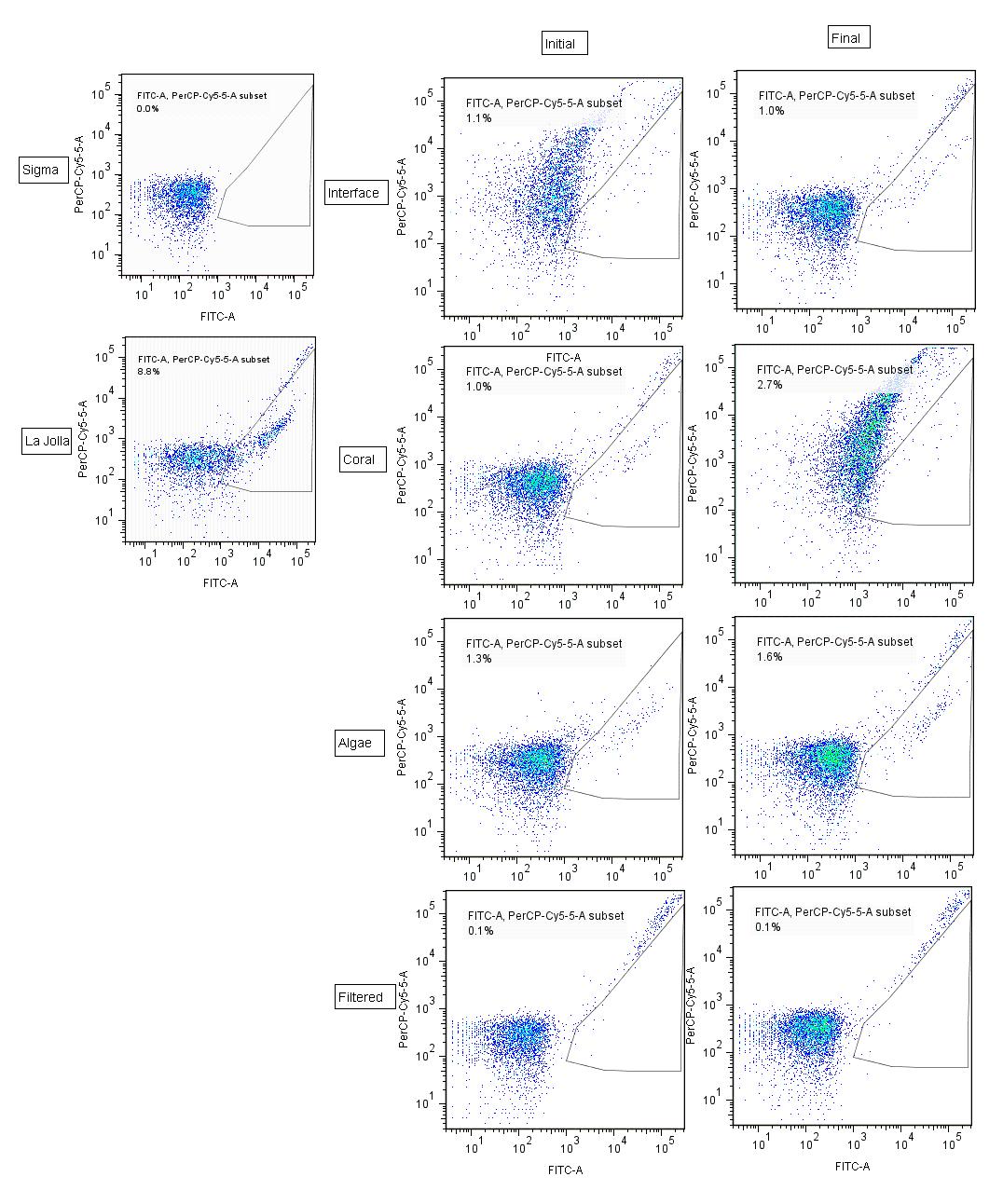

Supplement: Data S18 — Raw data of stained samples exported from the flow cytometer BD FACS-Canto and analyzed in Figs. 3 and 4. [file peerj-05-3423-s023.jpg]
