# Supplementary material for: Microbial bioenergetics of coral-algal interactions
Source: PeerJ. 2017 Jun 21;5:e3423. doi: 10.7717/peerj.3423 (PMC5482263; doi:10.7717/peerj.3423)
Supplement: Table S1 [file peerj-05-3423-s002.docx]

Table S1:
Statistical output of one-way ANOVA and subsequent Student t-test *post hoc* analysis for power output (μW) normalized by weight of the sample (g).

| **Normalized power output by treatment** | **ANOVA p value: <0.0001** |  |  |  |  |
| --- | --- | --- | --- | --- | --- |
| **Source** | **Degrees of Freedom** | **Sum of Squares** | **Mean Square** | **F Ratio** | **Probability > F** |
| Sample | 3 | 0.07082148 | 0.023607 | 10.7653 | <.0001 |
| Error | 25 | 0.05482219 | 0.002193 |  |  |
| C. Total | 28 | 0.12564367 |  |  |  |
|  |  |  |  |  |  |
| **Treatment** | **Mean (μW/g)** | **Standard error (μW/g)** |  |  |  |
| interface | 33.6528 | 1.656 |  |  |  |
| coral | 28.5528 | 1.656 |  |  |  |
| algae | 26.5341 | 1.77 |  |  |  |
| water | 19.4359 | 1.912 |  |  |  |
|  |  |  |  |  |  |
| **Pair wise treatment t-tests** | ***post hoc* t-test p value** |  |  |  |  |
| interface-coral | 0.039 |  |  |  |  |
| interface-algae | 0.007 |  |  |  |  |
| coral-algae | 0.4128 |  |  |  |  |
| water-coral | 0.0014 |  |  |  |  |
| water-algae | 0.0116 |  |  |  |  |
| water- interface | <0.0001 |  |  |  |  |
