# Supplementary material for: Microbial bioenergetics of coral-algal interactions
Source: PeerJ. 2017 Jun 21;5:e3423. doi: 10.7717/peerj.3423 (PMC5482263; doi:10.7717/peerj.3423)
Supplement: Table S2 [file peerj-05-3423-s003.docx]

Table S2:
Statistical output of one-way ANOVA and subsequent Student t-test *post hoc* analysi*s* for heterotroph: autotroph ratios.

| **Heterotroph: autotroph ratio by treatment** | **ANOVA p value: 0.0345** |  |  |  |  |
| --- | --- | --- | --- | --- | --- |
| **Source** | **Degrees of Freedom** | **Sum of Squares** | **Mean Square** | **F Ratio** | **Probability > F** |
| Sample | 3 | 1338.5844 | 446.195 | 3.4106 | 0.0345 |
| Error | 23 | 3008.9599 | 130.824 |  |  |
| C. Total | 26 | 4347.5444 |  |  |  |
|  |  |  |  |  |  |
| **Treatment** | **Mean** | **Standard error** |  |  |  |
| interface | 32.946 | 4.3231 |  |  |  |
| coral | 18.4932 | 4.6695 |  |  |  |
| algae | 15.6103 | 4.3231 |  |  |  |
| water | 27.8524 | 4.3231 |  |  |  |
|  |  |  |  |  |  |
| **Pair wise treatment t-tests** | ***post hoc* t-test p value** |  |  |  |  |
| interface-coral | 0.0328 |  |  |  |  |
| interface-algae | 0.094 |  |  |  |  |
| coral-algae | 0.6548 |  |  |  |  |
| water-coral | 0.1549 |  |  |  |  |
| water-algae | 0.0572 |  |  |  |  |
| water- interface | 0.4133 |  |  |  |  |
