# Supplementary material for: Microbial bioenergetics of coral-algal interactions
Source: PeerJ. 2017 Jun 21;5:e3423. doi: 10.7717/peerj.3423 (PMC5482263; doi:10.7717/peerj.3423)
Supplement: Table S3 [file peerj-05-3423-s004.docx]

Table S3:
Statistical output of one-way ANOVA and subsequent Student t-test *post hoc* analysi*s* for dissolved oxygen concentration (μM).

| **Oxygen concentration by treatment** | **ANOVA p value: 0.2827** |  |  |  |  |
| --- | --- | --- | --- | --- | --- |
| **Source** | **Degrees of Freedom** | **Sum of Squares** | **Mean Square** | **F Ratio** | **Probability > F** |
| Sample | 3 | 7371.608 | 2457.2 | 1.4054 | 0.2827 |
| Error | 14 | 24478.04 | 1748.43 |  |  |
| C. Total | 17 | 31849.648 |  |  |  |
|  |  |  |  |  |  |
| **Treatment** | **Mean (μM)** | **Standard error (μM)** |  |  |  |
| interface | 39.8299 | 18.7 |  |  |  |
| coral | 82.4222 | 20.907 |  |  |  |
| algae | 63.4961 | 20.907 |  |  |  |
| water | 90.3468 | 18.7 |  |  |  |
|  |  |  |  |  |  |
| **Pair wise treatment t-tests** | ***post hoc* t-test p value** |  |  |  |  |
| interface-coral | 0.1512 |  |  |  |  |
| interface-algae | 0.413 |  |  |  |  |
| coral-algae | 0.5324 |  |  |  |  |
| water-coral | 0.7817 |  |  |  |  |
| water-algae | 0.3547 |  |  |  |  |
| water- interface | 0.0768 |  |  |  |  |
