# Supplementary material for: Microbial bioenergetics of coral-algal interactions
Source: PeerJ. 2017 Jun 21;5:e3423. doi: 10.7717/peerj.3423 (PMC5482263; doi:10.7717/peerj.3423)
Supplement: Table S4 [file peerj-05-3423-s005.docx]

Table S4:
Statistical output of one-way ANOVA and subsequent Student t-test *post hoc* analysi*s* for total cellular abundance (cells/ mL) .

| **Cell abundance by treatment** | **ANOVA p value: 0.2827** |  |  |  |  |
| --- | --- | --- | --- | --- | --- |
| **Source** | **Degrees of Freedom** | **Sum of Squares** | **Mean Square** | **F Ratio** | **Probability > F** |
| Sample | 3 | 1.24E+13 | 4.12E+12 | 0.5631 | 0.6444 |
| Error | 25 | 1.83E+14 | 7.32E+12 |  |  |
| C. Total | 28 | 1.95E+14 |  |  |  |
|  |  |  |  |  |  |
| **Treatment** | **Mean (cells/ mL)** | **Standard error (cells/ mL)** |  |  |  |
| interface | 3290593 | 956369.6416 |  |  |  |
| coral | 3043806 | 956369.6416 |  |  |  |
| algae | 2929968 | 956369.6416 |  |  |  |
| water | 1398291 | 1209722.541 |  |  |  |
|  |  |  |  |  |  |
| **Pair wise treatment t-tests** | ***post hoc* t-test p value** |  |  |  |  |
| interface-coral | 0.8567 |  |  |  |  |
| interface-algae | 0.7919 |  |  |  |  |
| coral-algae | 0.9336 |  |  |  |  |
| water-coral | 0.2961 |  |  |  |  |
| water-algae | 0.3301 |  |  |  |  |
| water- interface | 0.2312 |  |  |  |  |
